# Prevalence of Allergic Rhinitis Among Children and Its Characteristics in Al-Qassim Region, Saudi Arabia

**DOI:** 10.7759/cureus.80835

**Published:** 2025-03-19

**Authors:** Saleh F Aldubayyan, Rabab A Alswyan, Waleed A Alhazmi, Yazeed K Alhabeeb, Abdulelah M Alrubayan, Abdulrahman F Alsowinea

**Affiliations:** 1 Department of Otolaryngology, Head and Neck Surgery, Qassim Health Cluster, Buraydah, SAU; 2 Department of Otolaryngology, Head and Neck Surgery, Qassim University, Buraydah, SAU; 3 College of Medicine, Qassim University, Buraydah, SAU; 4 Department of Pediatrics, Qassim Health Cluster, Buraydah, SAU

**Keywords:** adolescents and ar, al-qassim region, ar prevalence, ent medical records, environmental factors, family history of allergies, isaac questionnaire, parental smoking, pediatric allergic rhinitis, saudi arabia

## Abstract

Objective

The objective of the study is to estimate the prevalence of allergic rhinitis (AR) and its characteristics among the pediatric population at Dr. Suliman AlHabib Hospital in Buraydah, Al-Qassim region, Saudi Arabia.

Methods

This cross-sectional, retrospective study utilized data (n = 217) from the ENT department’s medical records at Dr. Suliman AlHabib Hospital in Buraydah, covering a three-year period from January 1, 2020, to January 1, 2023. A modified version of the International Study of Asthma and Allergies in Children (ISAAC) questionnaire was employed. The data was analyzed using R language version 4.3.3 (R Foundation for Statistical Computing, Vienna, Austria).

Results

The prevalence of AR among the children studied was 7%, with male patients showing a higher prevalence than female patients. No significant correlation was found between AR prevalence and age or body mass index (BMI), although adolescents were noted to have the highest prevalence compared to other age groups. Family history of allergies and parental smoking emerged as potential influencing factors, suggesting that environmental and genetic components might play a role in the development of AR.

Conclusion

The study highlights key trends in the epidemiology of AR among children in Buraydah. Although several risk factors, such as family history and parental smoking, were identified, the findings suggest the need for further research to fully understand the underlying causes and influences on AR. Future studies should aim to expand the sample size and investigate these associations more comprehensively to support effective public health strategies in the region.

## Introduction

Allergic rhinitis (AR) is a common atopic condition affecting 10%-25% of the global population, impairing quality of life, and frequently coexisting with asthma and dermatitis [1]. AR has multifactorial origins, including genetics, allergens, and environmental factors, without a singular cause [2-5]. AR is characterized by symptoms such as nasal congestion, sneezing, and itching, triggered by allergens like pollen, dust, and pet dander. The symptoms are further exacerbated by factors such as pollution and climate change [2,6-8]. AR is triggered by the body's immune response to various allergens, leading to histamine overproduction and nasal inflammation [9]. The prevalence of AR has risen globally, particularly among children, with symptoms often manifesting before the age of six [8,10]. Diagnosis involves clinical evaluation and allergen testing, and the first line of treatment typically includes antihistamines and intranasal corticosteroids [11].

The Allergic Rhinitis and its Impact on Asthma (ARIA) non-governmental organization (NGO) develops guidelines and updates on management. Their research and revised guidelines in 2016/2017 showed that AR affects 10%-40% of the global population. In recent years, the prevalence of AR has drastically increased, with a range of 2%-25%. This condition negatively impacts individuals' quality of life due to both irritative symptoms and incurred medical expenses [12].

The studies conducted across multiple regions aimed to determine the prevalence of asthma and AR in children exposed to pets [13] and environmental factors [14]. Meta-analyses of 14 studies revealed asthma prevalence among children exposed to pets ranging from 13.3% to 24.7%, with a higher prevalence of 25.5% for AR. Another study concluded that particulate matter (PM10) exposure significantly increased rhinitis risk in children, highlighting the impact of animal and environmental-based allergens [14]. In Iran, the prevalence of AR was found to be 18% in children and 25% in adults [15]. European research identified a high prevalence of AR among children with otitis media with effusion, particularly due to dust mites [16]. A cross-sectional study in Bangkok, Thailand, linked AR prevalence to family history, paracetamol use, and pet exposure [17]. Taiwan reported a 42.8% AR prevalence among first graders, with pet cat ownership as a significant risk factor [18]. In Angola, a study found eczema, asthma, and AR prevalent among schoolchildren, with maternal smoking and antibiotic use as risk factors [19]. A study in Qatar revealed high prevalence rates for asthma, AR, and eczema [20], suggesting a need for further research into contributing factors. In Guangzhou, China, allergen sensitization was assessed in children with AR, showing a high positivity rate for inhaled allergens [21]. These findings underscore the need for targeted interventions and public awareness efforts to address the rising prevalence of asthma and AR among children globally.

In light of these findings, the present study aims to estimate the prevalence of AR among children at Dr. Suliman AlHabib Hospital in Buraydah, Al-Qassim region, Saudi Arabia. Additionally, this research will assess the sociodemographic features, comorbid conditions, and complications associated with AR while comparing the prevalence rates in the Al-Qassim region to other areas within the Kingdom of Saudi Arabia and global figures. Despite the growing recognition of AR as a significant health concern, regional data remain limited, particularly in pediatric populations. By addressing this gap, the study aims to provide essential epidemiological insights that can inform targeted interventions and improve patient outcomes.

## Materials and methods

Study design

This retrospective cross-sectional study involved a comprehensive review of medical charts for all pediatric patients diagnosed with AR between January 1, 2020, and January 1, 2023, at Dr. Suliman Alhabib Hospital in Buraydah, Al-Qassim region, Saudi Arabia. The study design aimed to assess the prevalence, associated factors, and outcomes of AR within a defined timeframe.

Study population and sample size

The study population comprised all pediatric patients diagnosed with AR during the specified period. Data collection included both male and female patients within this timeframe. Patients with incomplete medical records were excluded from the analysis to ensure the quality and completeness of data.

The inclusion criteria include all pediatric patients diagnosed with AR from January 1, 2020, to January 1, 2023. For the exclusion criteria, all patients with incomplete medical records were excluded to avoid biases and inaccuracies in data analysis.

Study measures

Data were extracted from hospital records and securely stored. The primary data included (1) demographic information such as age, gender, and residency; (2) diagnosis details including the AR diagnosis date, associated comorbidities, and treatment history; (3) symptom characteristics like onset, duration, and severity of AR symptoms; (4) associated factors including environmental exposures (e.g., pets and pollution) and potential triggers; and (5) medical history pertaining to family history of allergies and asthma.

Data collection involved the retrospective retrieval of patients' medical records from the hospital. All collected data were entered into an Excel sheet (Microsoft Corp., Redmond, WA, US) and stored securely on a password-protected laptop to ensure confidentiality and data integrity.

Ethics statement

Ethical approval was obtained from the Institutional Review Board at Dr. Suliman Alhabib Hospital (RC23.09.13). The study adhered to ethical guidelines to ensure the privacy of collected data. Personal information was not disclosed in any publications; only a summary of the statistical findings was presented. The researchers ensured that only anonymized data were used in the analysis.

Statistical analysis

Descriptive statistics were employed for continuous and categorical data using R language version 4.3.3 (R Foundation for Statistical Computing, Vienna, Austria) [22]. The dataset had less than 5% missing body mass index (BMI) data, prompting the use of multiple imputations to handle the missing values. Median imputation was chosen due to potential skewness and variations in growth patterns in children's BMI data. This method offered a robust estimate of central tendency, especially in the presence of outliers, ensuring that the imputed values accurately reflected typical BMI values for children. The association between variables was assessed using chi-squared and t-tests, with statistical significance set at a p-value < 0.05.

## Results

**Figure 1:**
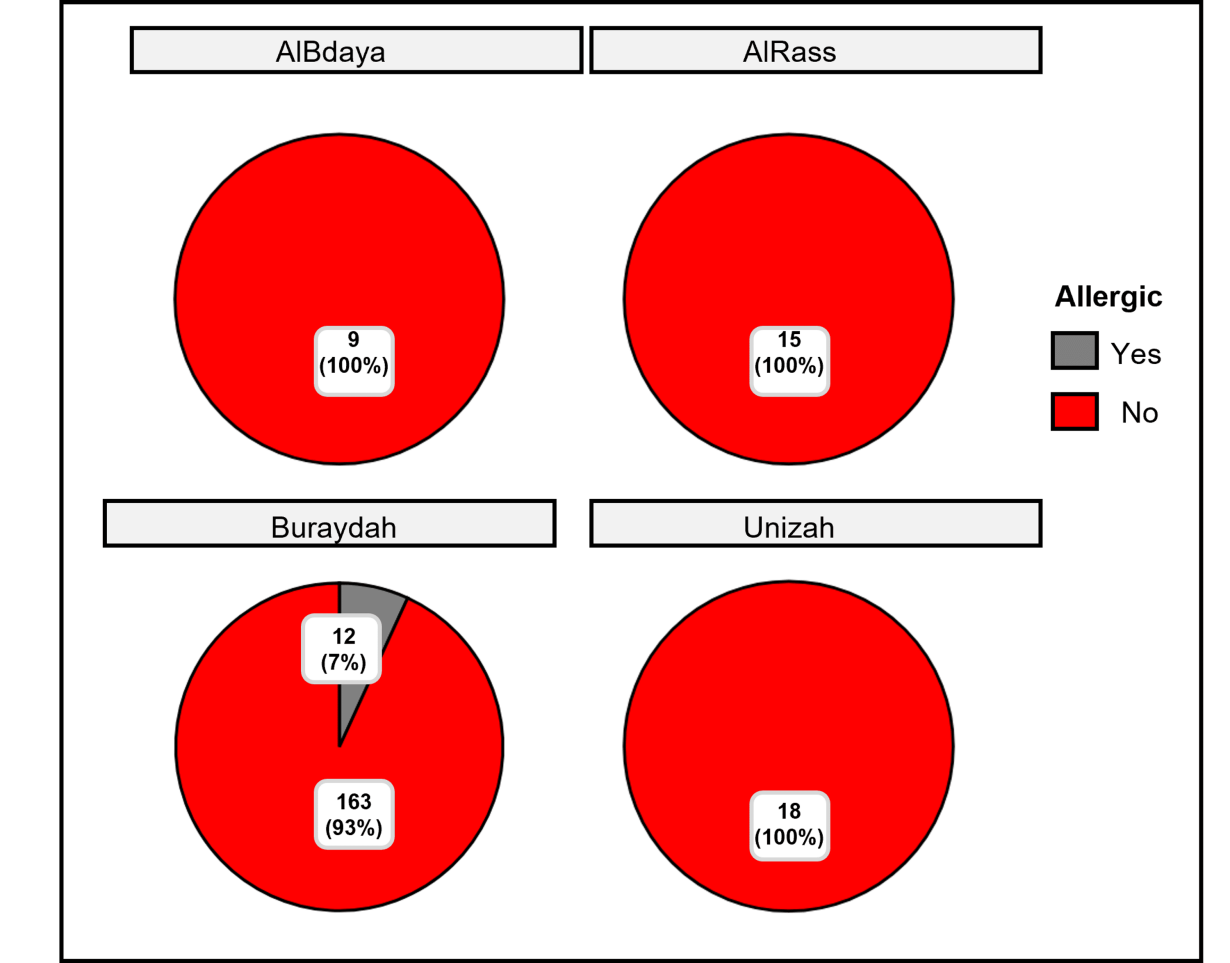
Prevalence of rhinitis among children in four cities: Albdaya, Alrass, Buraydah, and Unizah

Figure 1 illustrates the prevalence of AR among children with allergies in four cities. In Albdaya and Alrass, nine (100%) and 15 (100%) children, respectively, did not report AR, as determined by the chi-squared goodness-of-fit test (p =0.003 for Albdaya and p < 0.001 for Alrass). In Buraydah, 163 (93.1%) children out of 175 did not have AR, while 12 (6.9%) reported it (χ²_gof_ = 130.29, p < 0.001). Similarly, all 18 (100%) children in Unizah did not report AR, with a significant result (χ²_gof_ = 18, p < 0.001). Cramér’s V statistic indicated a negligible association between AR prevalence and location (V = 0.01), and the Bayesian Gunel-Dickey test statistic (a = 1.00) suggests a strong model fit. Overall, most children in these cities did not report AR, with a small exception in Buraydah (Table 1).

**Table 1:** Statistical analysis of allergic rhinitis (AR) prevalence among children across different demographic and categorical variables The table summarizes chi-squared values (χ²_gof_), p-values, Cramér’s V effect size, and 95% confidence intervals (CIs) for Cramér’s V, along with sample sizes and proportions of children reporting AR ("Yes") or not reporting AR ("No"). The data is categorized by location, gender, age group, education level, residence, family history of AR, and parental smoking status. CI: confidence interval

Parameter	Sample size (n)	Yes (%)	No (%)	Cramér’s V	95% CI for Cramér’s V	Chi-squared goodness-of-fit (χ²_gof_)	p-value
Location							
Albdaya	9	0 (0%)	9 (100%)	-	-	9	0.003
Alrass	15	0 (0%)	15 (100%)	-	-	15	p < 0.001
Buraydah	175	12 (7%)	163 (93%)	0.01	(0.00, 0.19)	130.29	p < 0.001
Unizah	18	0 (0%)	18 (100%)	-	-	18	p < 0.001
Gender							
Females	62	1 (2%)	61 (98%)	-	-	58.06	p < 0.001
Males	113	11 (10%)	102 (90%)	-	-	73.28	p < 0.001
Age group							
Adolescents	62	6 (10%)	56 (90%)	-	-	40.32	p < 0.001
Preschoolers	12	1 (8%)	11 (92%)	-	-	8.33	0.003
School-age children	96	5 (5%)	91 (95%)	-	-	77.04	p < 0.001
Toddlers	5	0 (0%)	5 (100%)	-	-	5	0.03
Education levels							
Primary and below	122	10 (8%)	112 (92%)	-	-	85.28	p < 0.001
Secondary and below	53	2 (4%)	51 (96%)	-	-	45.3	p < 0.001
Residence							
Buraydah residents	175	12 (7%)	163 (93%)	0.1	(0.00, 1.00)	130.29	p < 0.001
Non-Buraydah residents	42	0 (0%)	42 (100%)	0.1	(0.00, 1.00)	42	p < 0.001
Family history of allergic rhinitis							
No	99	5 (5%)	94 (95%)	0.03	(0.00, 1.00)	80.01	p < 0.001
Yes	76	7 (9%)	69 (91%)	0.03	(0.00, 1.00)	50.58	p < 0.001
Family history of smoking							
No	120	7 (6%)	113 (94%)	0	(0.00, 1.00)	93.63	p < 0.001
Unknown	34	3 (9%)	31 (91%)	0	(0.00, 1.00)	23.06	p < 0.001
Yes	21	0 (0%)	42 (100%)	0	(0.00, 1.00)	13.76	p < 0.001

**Figure 2:**
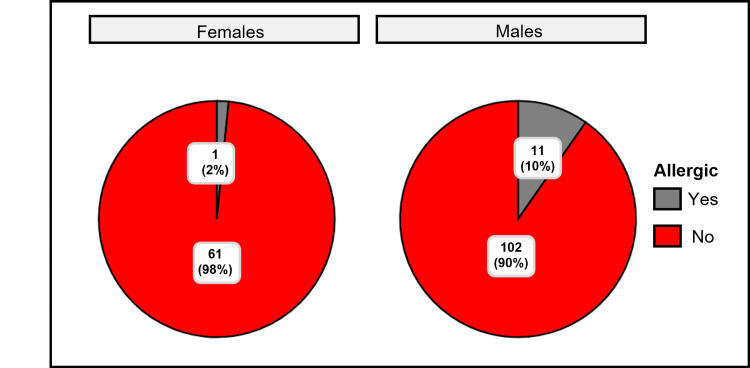
Prevalence of allergic rhinitis (AR) among male and female children in the Al-Qassim region

There is a statistically significant association between gender (female vs. male) and allergic status among children with AR in Buraydah, as indicated by the chi-squared Pearson test (χ²_Pearson_ = 4.13, p = 0.04). Cramér’s V statistic (0.13) suggests a small to moderate association, showing that allergic status varies between genders (Table 1, Figure 2). This result provides strong evidence against the null hypothesis.

**Figure 3:**
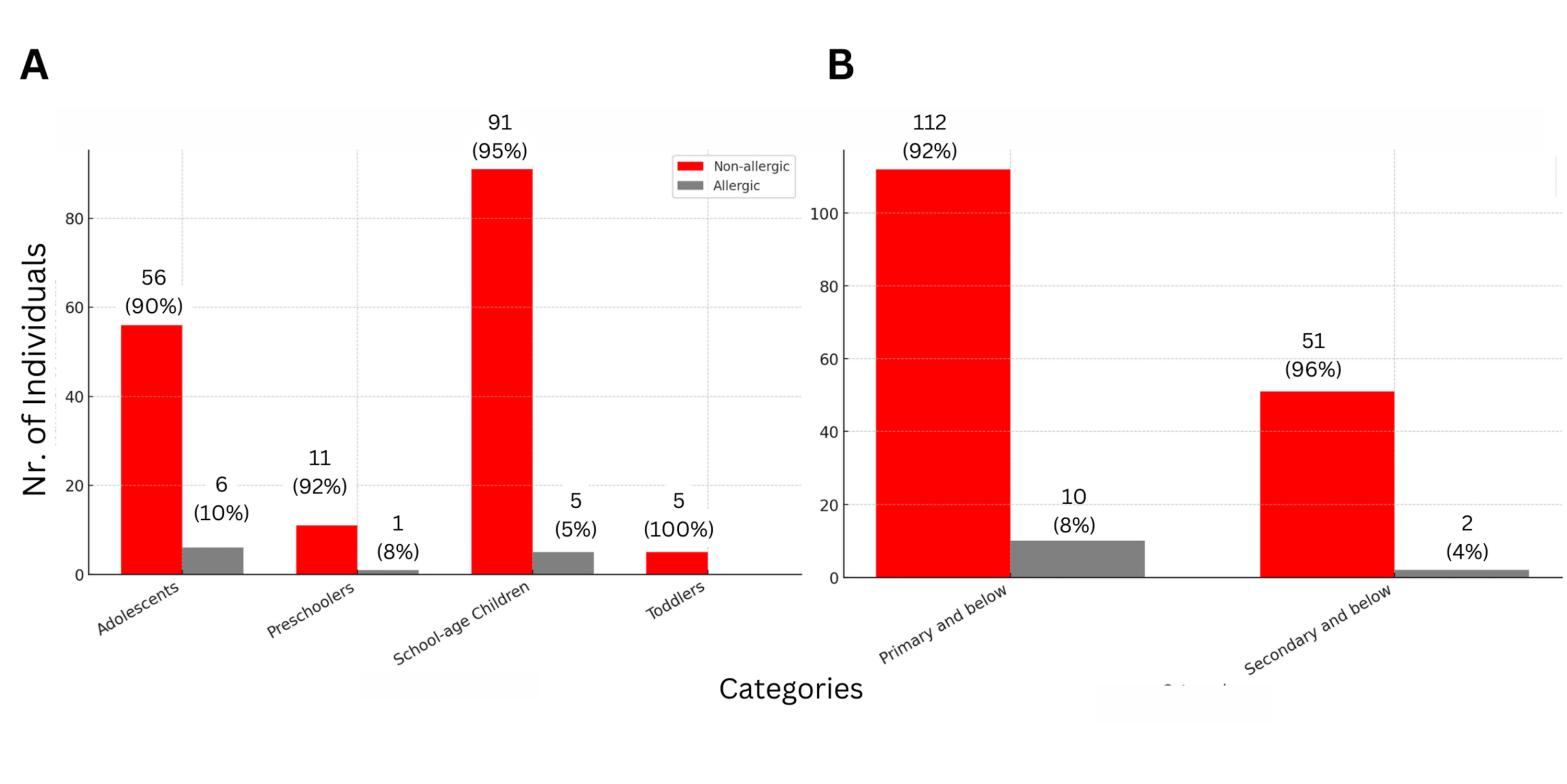
Prevalence of AR among children categorized by (A) age groups and (B) education level (primary and below vs. secondary and above)

Figure 3A illustrates the prevalence of AR among children with allergies, categorized into age groups (toddlers, preschoolers, school-age children, and adolescents). No statistically significant differences were observed between the age groups, though significant differences were found within the age groups themselves.

Figure 3B examines the relationship between education level ("Primary and below" vs. "Secondary and above") and allergic status among children with AR. The chi-squared test (χ²_Pearson_ = 1.13, p = 0.29) indicates no statistically significant association between education level and allergic status.

Within education levels, significant differences were observed. Among the "Primary and below" group, 10 (8%) children were allergic, while 112 (92%) were non-allergic (χ²_gof_ = 85.28, p < 0.001). Similarly, in the "Secondary and above" group, two (4%) children were allergic, and 51 (96%) were non-allergic (χ²_gof_ = 45.3, p < 0.001).

While within-group differences for each education level were statistically significant, overall differences between education levels were not. The chi-squared test (χ²_Pearson_ = 1.13, p = 0.29) confirms no significant association between education level and allergic status, and Cramér’s V statistic (0.03) suggests a very weak association.

The BMI of allergic and non-allergic children with AR in Buraydah was compared using Welch’s t-test (t = 1.18, p = 0.26), which indicated no statistically significant difference between the two groups. Overlapping BMI distributions support this conclusion, and the Bayes factor provides weak evidence against the null hypothesis.

Additionally, the relationship between age and BMI among children with AR in Buraydah was explored. The t-test (p = 0.94) revealed no significant difference between the means of age and BMI, suggesting that any observed differences are likely due to random variation rather than a true relationship between these variables.

**Figure 4:**
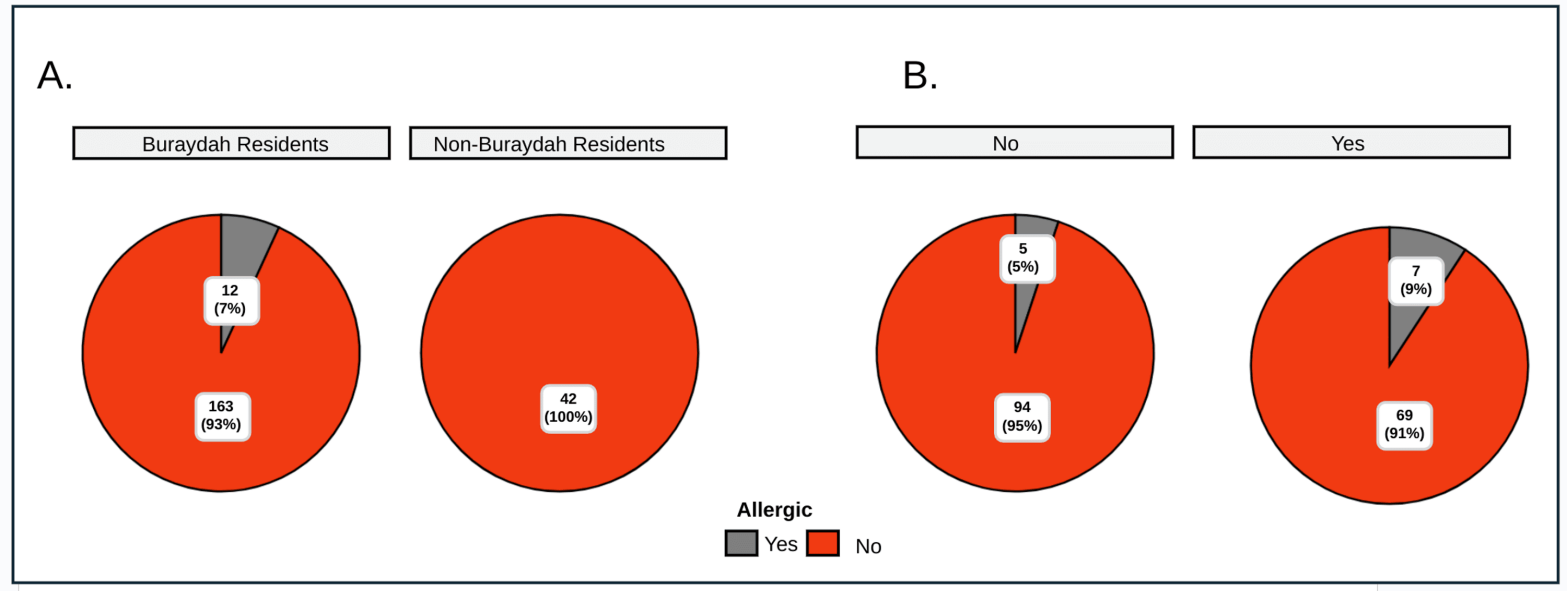
Relationship between (A) residence (Buraydah vs. non-Buraydah) and (B) family history of allergic rhinitis (yes or no) with allergic rhinitis (AR) status among children with rhinitis

Figure 4A explores the association between residence and allergic status among children with AR. The chi-squared test (χ²_Pearson_ = 3.05, p = 0.08) suggests no significant association between residence (Buraydah vs. non-Buraydah) and allergic status. Although the p-value is close to the significance threshold, Cramér’s V statistic (0.10) indicates a small to moderate association. Statistically significant within-group differences were observed in residence, but these were not statistically significant across groups.

The association between family history of AR (yes vs. no) and allergic status in children with AR in Buraydah was analyzed using a chi-squared test (χ² = 1.16, p = 0.28), which indicated no statistically significant association (Figure 4B). Among children without a family history of AR, five (5%) were allergic, while 94 (95%) were non-allergic, showing a statistically significant within-group difference (χ²_gof_ = 80.01, p < 0.001). Similarly, for children with a family history of AR, seven (9%) were allergic, and 69 (91%) were non-allergic, also demonstrating a significant within-group difference (χ²_gof_ = 50.58, p < 0.001). However, differences between the groups (family history vs. no family history) were not statistically significant.

**Figure 5:**
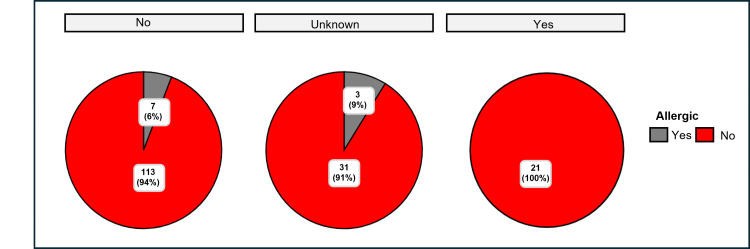
Relationship between family history of smoking (yes/unknown/no) with allergic rhinitis (AR) status among children with rhinitis

We further examined the association between parental smoking status and allergic status in children with AR in Buraydah (Figure 5). The chi-squared Pearson test (χ²_Pearson_ = 0.64, p = 0.73) suggests no significant association between the two variables, and Cramér’s V statistic (0.00) indicates a very weak association. However, significant within-group differences were observed among children based on parental smoking status.

## Discussion

The present study investigated AR among children residing in the Al-Qassim region of the Kingdom of Saudi Arabia. Age, gender, BMI, family history of rhinitis, education level, and parental smoking status were considered independent variables, while allergic status was regarded as the dependent variable. The study reported an AR prevalence of 7% (12/175), slightly lower than the 10.48% prevalence documented in a 2023 systematic review evaluating AR among children across 22 studies [11]. In Buraydah, 12 out of 175 (7%) children reported AR, while 163 (93%) children were non-allergic. Conversely, in Albdaya, Alrass, and Unizah, all of the children (9/9 in Albdaya, 15/15 in Alrass, and 18/18 in Unizah) did not report AR, yielding highly significant chi-squared results (Figure 1, Table 1). Cramér’s V revealed a negligible association between AR prevalence and location, reaffirming that most children in these cities do not report AR, with Buraydah being the sole exception (12/175, 7% reporting AR).

Gender emerged as the only variable significantly associated with allergic status among children affected by rhinitis (Figure 2). A chi-squared test revealed a statistically significant relationship (χ²_Pearson_ = 4.13, p = 0.04), with notable within-group disparities. Male children demonstrated a higher prevalence of AR; 11 male children (10%) were found to have AR as compared to only one female (2%).

The WHO categorizes age groups for children as follows: toddlers (1-3 years), preschoolers (3-5 years), school-age children (5-12 years), and adolescents (12-18 years). Although the current study did not yield statistical significance, it reported a high prevalence of AR among adolescents (six (10%)), followed by preschoolers (one (8%)), and then school-age children (five (5%)), as illustrated in Figure 3A. These findings closely resemble those of the study conducted by Adegbiji et al., which found a high incidence of AR among preschoolers. In their study at Nigeria's Ekiti State University Teaching Hospital, 265 out of 4,341 ENT patients had AR, yielding a 6.1% prevalence. Preschoolers aged 1-5 years had the highest occurrence (47.9%), and 54.9% had a positive family history [3]. Furthermore, the current study does not have sufficient evidence to conclude that education level and allergic status are related among children with AR in Buraydah (see Figure 3B).

The current study reports no significant correlation between the age and BMI of children in Buraydah afflicted with AR. The Pearson correlation coefficient signified a minimal linear association between age and BMI within this population, as shown in Figure 4B. While the findings lacked statistical significance (p = 0.94), the current study cannot definitively corroborate the findings of Almehizia et al. [23], conducted in Saudi Arabia, which reported that older age groups and overweight patients experienced more persistent forms of AR. In a June 2019 study in Saudi Arabia on AR, findings revealed underdiagnosis and undertreatment despite its high prevalence [24]. The current investigation lacked sufficient evidence to assert a correlation between familial rhinitis history and allergic status in children afflicted with rhinitis. However, it is noteworthy that there existed statistically significant intragroup disparities, as depicted in Figure 5B, suggesting that allergic status may yet be influenced by other variables within each subgroup.

Among children without a family history of AR, seven (6%) were allergic, while 113 (94%) were non-allergic (χ²_gof_ = 80.01, p <0.001), and among those with a family history, three (9%) were allergic, indicating significant within-group differences. Despite the absence of statistically significant differences in familial history across various categories, children exhibited a familial predisposition to AR. When compared to previous studies, it should be noted that this proportion is lower than the familial predisposition reported in 145 out of 265 children (54.7%) in the study by Adegbiji et al. Moreover, the investigation by Chinratanapisit et al. identified familial history as one of the risk factors for AR among children [17].

The current investigation lacks sufficient evidence to conclude if there is a relationship between parental smoking status and allergic status among children with rhinitis. However, it is noteworthy that statistically significant intragroup differences exist, as observed in Figure 5, suggesting that allergic status may still be influenced by other factors within each parental smoking status category. Thus, the present study cannot link parental smoking with AR, unlike the conclusions drawn by Arrais et al. [19], who identified maternal smoking as a risk factor for AR among children.

The present study examined the correlation between AR in children and the type of residence in which they reside. The findings, as illustrated in Figure 5A, indicated some suggestion of an association between residence (Buraydah vs. non-Buraydah) and allergic status among children with rhinitis, albeit not reaching statistical significance at conventional thresholds (χ²_Pearson_ = 3.05, p = 0.08). Cramér's V statistic revealed a small to moderate association, with the p-value slightly higher than 0.08. Hence, while there might be a potential link between residence and allergic status, further studies with a larger sample size are necessary.

Study limitations

The present study encountered several limitations that may impact the generalization of its findings. Initially, the dataset consisted of children from Buraydah (175), Alrass (15), Albdaya (9), and Unizah (18), rendering it inadequate for comparative analysis. To enhance comparative analysis capabilities, expanding data collection to include more participants from medical facilities in AlRass, Albdaya, and Unizah could be beneficial.

Additionally, the data on BMI was incomplete, although comprising less than 5% of the total dataset. To address this issue, multiple imputation was employed to complete missing values. It is recommended for future research to ensure complete datasets, by documenting the actual weights and heights of all children during triage, facilitating the computation of their BMI without missing records.

Furthermore, the study relied on self-reported data for AR and certain exposure variables, introducing the potential for recall bias. While structured questionnaires were used to standardize responses, some degree of recall bias remains unavoidable. Future studies may benefit from incorporating objective diagnostic measures or cross-referencing medical records to minimize this limitation.

Lastly, the possibility of confounding cannot be entirely ruled out. While key variables such as age, gender, and BMI were considered, other unmeasured factors, including genetic predisposition, environmental pollutants, and household allergen exposure, may have influenced the findings.

## Conclusions

In conclusion, our study in four different cities of the Al-Qassim region in Saudi Arabia explored factors influencing AR among children. The study found a 7% prevalence of AR, with male children showing a higher prevalence. While age and BMI showed no significant correlation, adolescents exhibited the highest prevalence. Familial history and parental smoking may influence allergic status, though further research is necessary. The investigation into the residence type suggested a potential association with allergic status, requiring additional study. The findings highlight important trends in AR epidemiology among children. Continued research is essential to fully understand these dynamics and inform effective public health strategies.
